# Finite-Time Interactive Control of Robots with Multiple Interaction Modes

**DOI:** 10.3390/s22103668

**Published:** 2022-05-11

**Authors:** Jiantao Yang, Tairen Sun

**Affiliations:** The School of Health Science and Engineering, University of Shanghai for Science and Technology, Shanghai 200093, China; jty@usst.edu.cn

**Keywords:** multiple interaction modes, finite-time control, human–robot interaction, impedance control, trajectory tracking

## Abstract

This paper proposes a finite-time multi-modal robotic control strategy for physical human–robot interaction. The proposed multi-modal controller consists of a modified super-twisting-based finite-time control term that is designed in each interaction mode and a continuity-guaranteed control term. The finite-time control term guarantees finite-time achievement of the desired impedance dynamics in active interaction mode (AIM), makes the tracking error of the reference trajectory converge to zero in finite time in passive interaction mode (PIM), and also guarantees robotic motion stop in finite time in safety-stop mode (SSM). Meanwhile, the continuity-guaranteed control term guarantees control input continuity and steady interaction modes transition. The finite-time closed-loop control stability and the control effectiveness is validated by Lyapunov-based theoretical analysis and simulations on a robot manipulator.

## 1. Introduction

With the development of modern robots, human robot co-existent scenario are proven to be a clear trend, which gives rise to the emerging field of research named human robot interaction (HRI). Human collaborated with robots will enhance their strength and efficiency. When maximizing the performance, efficiency, and applicability of coupled human–robot systems, robots are used for assisting users toward realizing their planed action makes up of half of the solution, and the other half is ensuring the reliability and safety of the system [1]. Today, robot technology can be seen everywhere, including industrial robot, social robot, medical robot. It seems that robot technology can do anything. However, a serious problem also arises that robots’ reliability may be overtrusted [2]. For example, it is reported that people may copy Artificial Intelligence (AI)’ action without evaluations [3]. Thus, reliability and safety in human–robot interaction should be investigated widely.

In active interaction mode (AIM) of physical human–robot interaction, the human initiates a motion and the robot plays as a follower. In this mode, interaction compliance is important to guarantee the human’s comfort and safety. As a powerful active compliance control approach, impedance control proposed in 1980s by Hogan can regulate robot impedance in certain ranges through the desired impedance dynamics which describes a dynamical relationship between robot position and interaction force [4]. Since its first development, it has received large much research attention and applied in service robots and industrial robots [5,6,7]. Robot modeling uncertainties are the main factor that affects impedance control stability and robustness. To improve control robustness, categories of impedance control strategies were proposed based on adaptive control [8,9,10,11], neural networks [12,13,14,15,16], sliding mode technique [17,18], and iterative learning [19,20]. However, these impedance controllers obtain infinite-time control stability and the desired impedance dynamics in these results were achieved in infinite time. Compared with infinite-time control, finite-time control of robot has better control robustness and can make robot track the desired trajectory in finite time [21]. Designing finite-time impedance control is of significant value for human–robot interaction.

The sliding mode is a possible technique to be used for finite-time impedance control. In [22], a sliding-mode impedance controller was proposed with the switching function reaching zero in finite time. After this finite time, the switching function remains constant at zero, and the desired surface can be theoretically achieved. However, chattering severely affects the impedance control performance and achievement of the desired impedance dynamics. In [23], a dead-zone strategy was applied to alleviate chattering problem in a sliding-mode impedance controller. However, this strategy may not effectively decrease chattering and may lead to difficulties in arriving at the desired sliding surface, which will affect the impedance control performances. Super-twisting algorithm is a category of sliding mode control without causing severe chattering problem and can be considered as a possible good choice to design finite-time impedance control.

Besides AIM, there exist some other human–robot interaction modes, including passive interaction mode (PIM) and safety-stop mode (SSM). In PIM, there is no human active motion and the robot moves the human to follow a prescribed trajectory, while the robot should move the human to stop at a certain position quickly in order to avoid possible injuries in SSM. The existing physical human–robot interaction control strategies were developed mainly in AIM or PIM. In the case of multiple interaction modes coexistence, interaction mode switches along with jumps of the reference trajectories and interaction forces which may lead to control input discontinuity and further lead to the chattering problem.

According to the above analysis, this paper proposes a finite-time multi-modal interactive controller. The finite-time closed-loop control stability is validated by Lyapunov-based theoretical analysis and the control effectiveness is illustrated by simulations on a two-link robot arm. Compared with the related results, the contributions of this paper include: (i) In AIM, a finite-time impedance control is designed based on a modified super-twisting algorithm, such that the impedance error converges to zero in finite time without causing chattering problem. (ii) A steady switch control term is designed to guarantee control continuity and steady interaction modes switch. (iii) The finite-time multi-modal control term guarantee the finite-time achievement of the desired impedance dynamics in AIM, make the tracking error of the reference trajectory converge to zero in finite time in PIM, and guarantee robotic motion stop in finite time in SSM.

## 2. Robot Dynamics

Consider the robot dynamics in the joint space in the following form

(1) $$\begin{array}{c} M\left(q\right)\ddot{q}+C\left(q,\dot{q}\right)\dot{q}+G\left(q\right)+F\dot{q}=\tau +\tau_{h} \end{array}$$

where $q\in R^{n}$ denotes the joint position, M(q) denotes the mass matrix, $C(q,\dot{q})$ denotes the Coriolis and centrifugal matrix, G(q) denotes the gravity torque, $F\dot{q}$ represents the friction torque with *F* being a constant matrix, $\tau_{h}$ and τ are the interaction force vector in joints and the system control input, respectively.

The matrices M(q), $C(q,\dot{q})$, and G(q) satisfy

(2) $$\begin{array}{cc} \; & M\left(q\right)=M_{0}\left(q\right)+\Delta M,C\left(q,\dot{q}\right)=C_{0}\left(q,\dot{q}\right)+\Delta C,\\ \; & G\left(q\right)=G_{0}\left(q\right)+\Delta G \end{array}$$

where $M_{0}\left(q\right)$, $C_{0}\left(q,\dot{q}\right)$, $G_{0}\left(q\right)$ are known matrices and ΔM,ΔC,ΔG are unknown terms.

**Property** **1.** *M(q) and $M_{0}\left(q\right)$ are symmetric and positive definite matrices and*

(3) $$\begin{array}{c} \sigma_{1}I\leq M_{0}\left(q\right)\leq \sigma_{2}I \end{array}$$

*where $\sigma_{1}$ and $\sigma_{2}$ are positive constants.*

**Property** **2.** *${\dot{M}}_{0}\left(q\right)-2C_{0}\left(q,\dot{q}\right)$ is skew symmetric, i.e.*

(4) $$\begin{array}{c} \xi^{T}\left({\dot{M}}_{0}\left(q\right)-2C_{0}\left(q,\dot{q}\right)\right)\xi =0,\forall \xi \in R^{n}. \end{array}$$

Based on (1) and (2), the robotic dynamics can be presented as

(5) $$\begin{array}{c} \ddot{q}=g\left(q,\dot{q}\right)+d+\tau +\tau_{h} \end{array}$$

where $g\left(q,\dot{q}\right)=-M_{0}{\left(q\right)}^{-1}C_{0}\left(q,\dot{q}\right)\dot{q}-M_{0}{\left(q\right)}^{-1}G_{0}\left(q\right)$, $d=-M_{0}^{-1}\left(\Delta M\ddot{q}+\Delta C\dot{q}+\Delta G+F\dot{q}\right)$ that satisfies $\left|\right|\dot{d}\left|\right|\leq d_{c}$.

## 3. Finite-Time Control Design

This section presents an adaptive controller for the considered robot to steady switches among multiple modes including active interaction mode (AIM), passive interaction mode (PIM), and safety-stop mode (SSM).

In the AIM, an adaptive admittance control strategy is required to realize the following desired impedance dynamics

(6) $$\begin{array}{c} M_{d}\left({\ddot{q}}_{d}-\ddot{q}\right)+B_{d}\left({\dot{q}}_{d}-\dot{q}\right)+K_{d}\left(q_{d}-q\right)=f_{h} \end{array}$$

where $M_{d}$, $B_{d}$, $K_{d}$ denote the desired inertia, the desired damping, and the desired stiffness, respectively; $q_{d}$ is the desired trajectory and satisfies $q_{d}^{\left(i\right)}\in L_{\infty },i=1,2,3$. To realize the desired impedance dynamics, a reference trajectory $q_{r}$ for the robot is designed as

(7) $$\begin{array}{c} M_{d}{\ddot{q}}_{r}+B_{d}{\dot{q}}_{r}+K_{d}q_{r}=-f_{h}+l_{d} \end{array}$$

where $l_{d}=M_{d}{\ddot{q}}_{d}+B_{d}{\dot{q}}_{d}+K_{d}q_{d}$. Define the admittance errors $e_{a}=q_{r}-q$ and $r_{a}={\dot{e}}_{a}+k_{a1}e_{a}$ whose dynamics satisfies

(8) $$\begin{array}{c} {\dot{r}}_{a}={\ddot{q}}_{r}+k_{a1}{\dot{e}}_{a}-g\left(q,\dot{q}\right)-d-\tau -\tau_{h}. \end{array}$$

In the PIM, the human has no motion intention and an adaptive control term should be designed for the robot to track the certain trajectory ${\bar{q}}_{r}$. Define the errors $e_{p}={\bar{q}}_{r}-q$ and $r_{p}={\dot{e}}_{p}+k_{p1}e_{p}$ whose dynamics satisfies

(9) $$\begin{array}{c} {\dot{r}}_{p}={\ddot{\bar{q}}}_{r}+k_{p1}{\dot{e}}_{p}-g\left(q,\dot{q}\right)-d-\tau -\tau_{h}. \end{array}$$

When ||f||≥R with *R* being a prior defined constant, possible injuries may occur to humans and the human–robot interaction should be stopped by triggering the SSM. Therefore, in this mode, the velocity $\dot{x}$ needs to converge to zero as quickly as possible.

**Remark** **1.** *Substituting (7) into the desired impedance dynamics (6), one can get*

(10) $$\begin{array}{c} M_{d}\left({\ddot{q}}_{r}-\ddot{q}\right)+B_{d}\left({\dot{q}}_{r}-\dot{q}\right)+K_{d}\left(q_{r}-q\right)=0. \end{array}$$

*Thus, the desired impedance dynamics can be achieved if $\lim_{t\to \infty }\left(q_{r}-q\right)=\lim_{t\to \infty }\left({\dot{q}}_{r}-\dot{q}\right)=\lim_{t\to \infty }\left({\ddot{q}}_{r}-\ddot{q}\right)=0$. The matrices $M_{d}$, $B_{d}$, and $K_{d}$ in (6) are usually chosen as positive definite, diagonal matrices. It should be noted that $q_{r}^{\left(i\right)}\in L_{\infty }$ for i=0,1,2,3 if ${\dot{f}}_{h}$ is bounded. The objective of this paper is to design a modified super-twisting-based finite-time controller, such that the unified error r converges to zero in finite time, where r is defined by*

$$\begin{array}{c} r=\left\{\begin{array}{cc} r_{a} & AIM\\ r_{p} & PIM\\ \dot{q} & SSM. \end{array}\right. \end{array}$$

**Remark** **2.** *It should be noted that in some applications more SSMs are requred, since the robot needs to stop at a desired position to keep human safety. For example, when muscular spasm happens in robot-assisted rehabilitation, the robot should help the related limbs move from a bent posture to a stretched posture or from a stretched posture to a bent posture.*

*To achieve the objectives of the multiple interaction modes and steady switches among these modes, we propose the following multi-modal adaptive controller*

(11) $$\begin{array}{c} \tau =S+\phi \end{array}$$

*where S is defined as*

(12) $$\begin{array}{c} S=\left\{\begin{array}{cc} S_{a}, & \mathrm{AIM}\\ S_{p}, & \mathrm{PIM}\\ S_{s}, & \mathrm{SSM} \end{array}\right. \end{array}$$

(13) $$\begin{array}{cc} S_{a}= & -\tau_{h}+{\ddot{q}}_{r}+k_{a1}{\dot{e}}_{a}-g\left(q,\dot{q}\right)+l_{a1}{\left|r_{a}\right|}^{0.5}\mathrm{sgn}\left(r_{a}\right)\\ \; & +l_{a2}r_{a}+\int_{0}^{t}\left(l_{a3}\mathrm{sgn}\left(r_{a}\left(\tau \right)\right)+l_{a4}r_{a}\left(\tau \right)\right)d\tau \end{array}$$

(14) $$\begin{array}{cc} S_{p}= & -\tau_{h}+{\ddot{\bar{q}}}_{r}+k_{p1}{\dot{e}}_{p}-g\left(q,\dot{q}\right)+l_{p1}{\left|r_{p}\right|}^{0.5}\mathrm{sgn}\left(r_{p}\right)\\ \; & +l_{p2}r_{p}+\int_{0}^{t}\left(l_{p3}\mathrm{sgn}\left(r_{p}\left(\tau \right)\right)+l_{p4}r_{p}\left(\tau \right)\right)d\tau \end{array}$$

(15) $$\begin{array}{cc} S_{s}= & -\tau_{h}-g\left(q,\dot{q}\right)-l_{s1}{\left|\dot{q}\right|}^{0.5}\mathrm{sgn}\left(\dot{q}\right)\\ \; & -l_{s2}\dot{q}-\int_{0}^{t}\left(l_{s3}\mathrm{sgn}\left(\dot{q}\left(\tau \right)\right)+l_{p4}\dot{q}\left(\tau \right)\right)d\tau \end{array}$$

*where the control gains satisfy*

(16) $$\begin{array}{cc} \; & l_{i1}>5^{0.25}d_{c}^{0.5},l_{i2}>0,l_{i3}>d_{c},\\ \; & l_{i4}>\frac{8l_{i2}^{2}l_{i3}+22l_{i2}^{2}d_{c}+9l_{i1}^{2}l_{i2}^{2}}{4l_{i3}-4d_{c}},i=a,p,s. \end{array}$$

*In (10), the control term ϕ is designed to guarantee steady interaction mode switches through the control input continuity and is specified as*

(17) $$\begin{array}{c} \dot{\phi }=-k_{1}\phi -k_{2}\mathrm{sgn}\left(\phi \right) \end{array}$$

*where $\phi \left(t_{k}\right)=S\left(t_{k}^{-}\right)-S\left(t_{k}\right)$.*

*$k_{1}$, $k_{2}$ are designed positive control gains, and $k_{1}>\max\left\{0.5/\lambda_{1},0.5/\lambda_{2}\right\}$.*

**Remark** **3.** *When multiple modes switches, the value jump of S possiblely leads to the control law τ being discontinuous. This may bring in robot vibration or undesirable human–robot interaction. The desgined control term ϕ guarantees the continuity of τ and the steady switches between the CIM and the SSM. Furthermore, ϕ can converge to zero in a predefined time. Thus, the transitional time in modes switches is controllable.*

**Theorem** **1.** *Design the multi-modal control scheme in (11) for the considered robot in (1), where the control parameters satisfy (16). Then, the desired impedance dynamics in (5) can be achieved through the finite-time convergence of $r_{a}$ when the AIM is active, the finite-time convergence of $e_{p}$ can be obtianed when the PIM is active, and the robot stops quickly through the finite-time convergence of $\dot{q}$ when the SSM is triggered. Furthermore, the designed control term ϕ in (10) guarantees steady mode switches.*

**Proof.**  When the AIM is active, the dynamics of $r_{a}={\left[r_{a1},\cdots ,r_{an}\right]}^{T}$ can be described as

(18) $$\begin{array}{cc} \; & {\dot{r}}_{ai}=-l_{a1}{\left|r_{ai}\right|}^{0.5}\mathrm{sgn}\left(r_{ai}\right)-l_{a2}r_{ai}+\rho_{i} \end{array}$$

(19) $$\begin{array}{cc} \; & {\dot{\rho }}_{i}=-l_{a3}\mathrm{sgn}\left(r_{ai}\right)-l_{a4}r_{ai}+{\dot{d}}_{i},i=1,2,\cdots ,n. \end{array}$$

Consider the following function

(20) $$\begin{array}{cc} V_{a}= & 2l_{a3}\left|r_{ai}\right|+l_{a4}r_{ai}^{2}+0.5\rho_{i}^{2}\\ \; & +0.5(l_{a1}|r_{ai}{{|}^{0.5}\mathrm{sgn}\left(r_{ai}\right)+l_{a2}r_{ai}-\rho_{i})}^{2}. \end{array}$$

which can presented as

(21) $$\begin{array}{c} V_{a}=\theta^{T}Q\theta \end{array}$$

with

(22) $$\begin{array}{cc} \; & \theta =\left[\right|r_{ai}{\left|\mathrm{sgn}\left(r_{ai}\right),r_{ai},\rho_{i}\right]}^{T}, \end{array}$$

(23) $$\begin{array}{cc} \; & Q=\frac{1}{2}\left[\begin{array}{ccc} 4l_{i3}+l_{i1}^{2} & l_{i1}l_{i2} & -l_{i1}\\ l_{i1}l_{i2} & 2l_{i4}+{ł}_{i2}^{2} & -l_{i2}\\ -l_{i1} & -l_{i2} & 2 \end{array}\right]>0. \end{array}$$

Taking the time derivative of $V_{a}$ and substituting (18) and (19) yields

(24) $$\begin{array}{c} {\dot{V}}_{a}=-\frac{1}{|r_{ai}{|}^{0.5}}\theta^{T}A\theta -\theta^{T}B\theta +D^{T}\theta \dot{d} \end{array}$$

where

(25) $$\begin{array}{cc} \; & A=\frac{l_{i1}}{2}\left[\begin{array}{ccc} 2l_{i3}+l_{i1}^{2} & 0 & -l_{i1}\\ 0 & 2l_{i4}+5l_{i2}^{2} & -3l_{i2}\\ -l_{i1} & -3l_{i2} & 1 \end{array}\right] \end{array}$$

(26) $$\begin{array}{cc} \; & B=l_{i2}\left[\begin{array}{ccc} l_{i3}+2l_{i1}^{2} & 0 & 0\\ 0 & l_{i4}+l_{i2}^{2} & -l_{i2}\\ 0 & -l_{i2} & 1 \end{array}\right] \end{array}$$

(27) $$\begin{array}{cc} \; & D={\left[-l_{i1},-l_{i2},2\right]}^{T}. \end{array}$$

The term $D^{T}\theta \dot{d}$ can be equivalently expressed as

(28) $$\begin{array}{cc} \; & D^{T}\theta \dot{d}=\theta^{T}E\theta /{\left|r_{ai}\right|}^{0.5}\\ \; & E=\left[\begin{array}{ccc} -l_{i1}\dot{d}\mathrm{sgn}\left(r_{ai}\right) & -0.5l_{i2}\dot{d}\mathrm{sgn}\left(r_{ai}\right) & \dot{d}\mathrm{sgn}\left(r_{ai}\right)\\ -0.5l_{i2}\dot{d}\mathrm{sgn}\left(r_{ai}\right) & 0 & 0\\ \dot{d}\mathrm{sgn}\left(r_{ai}\right) & 0 & 0 \end{array}\right] \end{array}$$

Substituting (28) into (24), one can obtain

(29) $$\begin{array}{c} {\dot{V}}_{a}=-\frac{1}{|r_{ai}{|}^{0.5}}\theta^{T}\left(A-E\right)\theta -\theta^{T}B\theta . \end{array}$$

Since $\left|\right|\dot{d}\left|\right|\leq d_{c}$ and the control gains satisfy (16), A−E and *B* are positive definite matrices. Then,

(30) $$\begin{array}{c} {\dot{V}}_{a}=-\frac{1}{|r_{ai}{|}^{0.5}}\lambda_{min}\left(A-E\right)\theta^{T}\theta -\lambda_{min}\left(B\right)\theta^{T}\theta \end{array}$$

where $\lambda_{min}\left(\cdot \right)$ denotes the minimum eigenvalue of a matrix.

From the definition of $V_{a}$ in (20),

(31) $$\begin{array}{c} |r_{ai}{|}^{0.5}\leq {\left||\theta |\right|}_{2}\leq V_{a}^{0.5}/\lambda_{min}^{0.5}\left(Q\right) \end{array}$$

Based on (29) and (30), one can obtain

(32) $$\begin{array}{c} {\dot{V}}_{ai}\leq -\lambda_{1}V_{a}^{0.5}-\lambda_{2}V_{a} \end{array}$$

where $\lambda_{1}=\lambda_{min}^{0.5}\left(Q\right)\lambda_{min}\left(A-C\right)/\lambda_{max}\left(Q\right)>0$ and $\lambda_{2}=\lambda_{min}\left(B\right)/\lambda_{max}\left(Q\right)>0$. Therefore, $V_{ai}$ and $r_{ai}$ for i=1,2,⋯,n converge to zero in finite time. □

Similar analysis can be conducted in PIM and SSM to conclude that $\lim_{t\to \infty }e_{p}=0$ if the PIM is active and $\lim_{t\to \infty }\dot{q}=0$ if the SSM is triggered. From Remark 3, the time-differentiable control term ϕ guarantees the continuity of the control law τ and ensures the steady transition of the multiple interaction modes.

**Figure 1:**
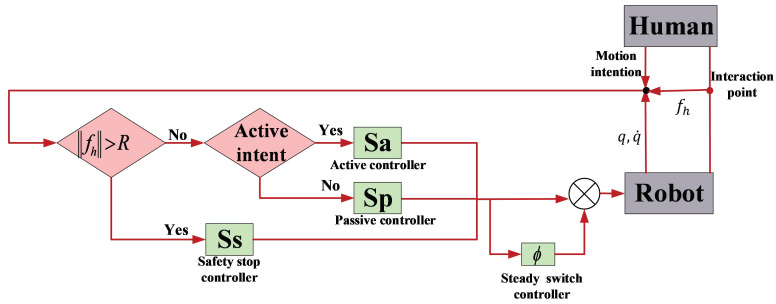
Schematic diagram of the controller.

**Remark** **4.** *The control design procedure is stated as follows. Step 1: Design active mode control, passive mode control, and safety stop mode control in each mode. Step 2: select multi-modal control according to the measured interactive force and human motion intention (see Figure 1). If the interactive force is bigger than a predefined threshold, the safety stop control is triggered. Otherwise, select active control when there is active human motion intention and select passive control when there is no obvious human motion intention. Step 3: If there exist interaction modes switches, the steady switch control is designed and combined with the control selected in Step 2 to construct the multi-modal control. At last, the designed multi-modal control is implemented on the considered robot.*

## 4. Simulation Results

To illustrate the effectiveness of the proposed adaptive multimodal control, simulations are taken on a two link robot manipulator with initial position and initial velocity $q\left(0\right)=\dot{q}\left(0\right)=0$. The manipulator’s two links have masses $m_{1}=2\;\mathrm{kg}$, $m_{2}=1.4\;\mathrm{kg}$, lengths $l_{1}=l_{2}=0.8\;m$, distances between their joints and respective center of masses $l_{c1}=l_{c2}=0.4\;m$, and moments of inertia $I_{1}=0.5$ kg·m${}^{2}$, $I_{2}=0.1$ kg·m${}^{2}$. The matrix *F* in (1) is defined by F=diag{0.3,0.3}. Denote $\theta_{1}=I1+I2+m1*Lc1*Lc1+m2*L1*L1+m2*Lc2*Lc2=2.04,\theta_{2}=m2*Lc2*Lc2+I2=0.3240,\theta_{3}=m2*L1*Lc2=0.4480,\theta_{4}=m1*Lc1+m2*L1=1.92,\theta_{5}=m2*Lc2=0.56,\theta_{6}=0.3,\theta_{7}=0.3$ and $\theta_{1}=I1+I2+m1*Lc1*Lc1+m2*L1*L1+m2*Lc2*Lc2=2.04,\theta_{2}=m2*Lc2*Lc2+I2=0.3240,\theta_{3}=m2*L1*Lc2=0.4480,\theta_{4}=m1*Lc1+m2*L1=1.92,\theta_{5}=m2*Lc2=0.56,\theta_{6}=0.3,\theta_{7}=0.3$.

The matrices M(q), $C(q,\dot{q})$, G(q), and *F* is defined by

(33) $$\begin{array}{cc} \; & M\left(q\right)=\left[\begin{array}{cc} \theta_{1}+2\theta_{3}\cos\left(q_{2}\right) & \theta_{3}\cos\left(q_{2}\right)+\theta_{2}\\ \theta_{3}\cos\left(q_{2}\right)+\theta_{2} & \theta_{2} \end{array}\right]\\ \; & C=\left[\begin{array}{cc} -\theta_{3}{\dot{q}}_{2}\sin\left(q_{2}\right) & -\theta_{3}\left({\dot{q}}_{1}+{\dot{q}}_{2}\right)\sin\left(q_{2}\right)\\ \theta_{3}{\dot{q}}_{1}\sin\left(q_{2}\right) & 0 \end{array}\right]\\ \; & G\left(q\right)={\left[\theta_{4}g\cos\left(q_{1}\right)+\theta_{5}g\cos\left(q_{1}+q_{2}\right),\theta_{5}g\cos\left(q_{1}+q_{2}\right)\right]}^{T}\\ \; & F=\mathrm{diag}\{\theta_{6},\theta_{7}\} \end{array}$$

and the known matrices $M_{0}\left(q\right)$, $C_{0}\left(q,\dot{q}\right)$, $G_{0}\left(q\right)$ are defined by replacing $\theta_{i}$, i=1,2,3,4,5 in M(q), $C(q,\dot{q})$, G(q) through $\theta_{i0}$, i=1,2,3,4,5, where $\theta_{10}=0.9962$, $\theta_{20}=0.1225$, $\theta_{30}=0.245$, $\theta_{40}=1.225$, and $\theta_{50}=0.35$.

In the simulation, suppose there exists active human motion intentions in t∈[0,8), there is no active human motion intention in t∈[8,15), and the interaction should be stopped at t=15 due to possible injuries. In t∈[0,8], the desired trajectory in (5) is $q_{d}={\left[0.6+0.3\cos\left(\pi t/10\right),0.6+0.3\sin\left(\pi t/10\right)\right]}^{T}$ and the desired impedance profiles are $M_{d}=I,B_{d}=8I,K_{d}=16I$. In t∈[8,15], the certain trajectory is ${\bar{q}}_{r}=\left[0.3\cos\left(\pi t/6\right),0.3\sin\left(\pi t/6\right)\right]$. In the interaction procedure, the interaction force is $\tau_{h}=J^{T}f_{h}$ with

(34) $$\begin{array}{c} f_{h}=\left\{\begin{array}{cc} {\left[5\sin\left(0.2t\right),5\cos\left(0.2t\right)\right]}^{T}, & t\in [0,8)\\ {\left[10\sin\left(0.2t\right),10\cos\left(0.2t\right)\right]}^{T}, & t\in [8,15)\\ {\left[20\exp\left(15-t\right),8\exp\left(15-t\right)\right]}^{T}, & t\in [15,20) \end{array}\right. \end{array}$$

The control parameters are chosen as $k_{a1}=k_{p1}=2$, $l_{a1}=l_{p1}=l_{s1}=3$, $l_{a2}=l_{p2}=l_{s2}=5$, $l_{a3}=l_{p3}=l_{s3}=8$, $l_{a4}=l_{p4}=l_{s4}=4$.

**Figure 2:**
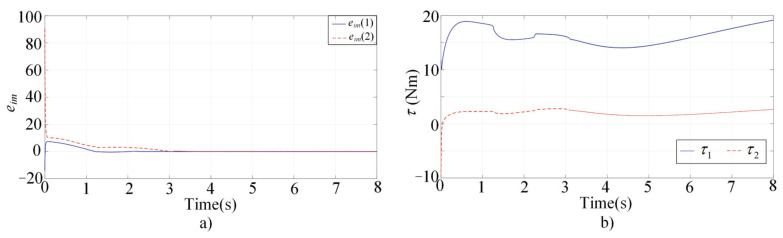
Simulation results of the multimodal controller in t∈[0,8). (**a**) The impedance errors; (**b**)The control inputs.

**Figure 3:**
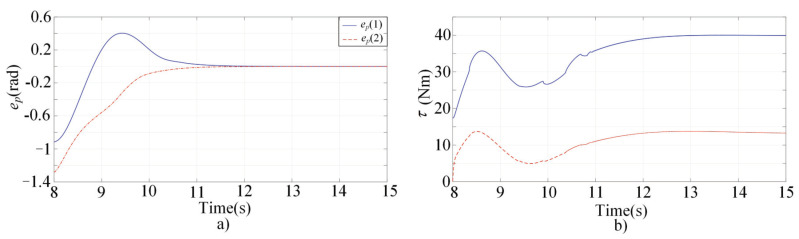
Simulation results of the multimodal controller in t∈[8,15). (**a**) The tracking errors; (**b**) The control inputs.

**Figure 4:**
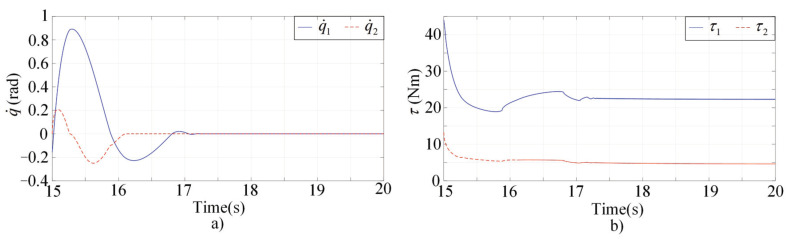
Simulation results of the multimodal controller in t∈[15,20]. (**a**) The convergence of the $\dot{q}$ to zero; (**b**) The control inputs.

**Figure 5:**
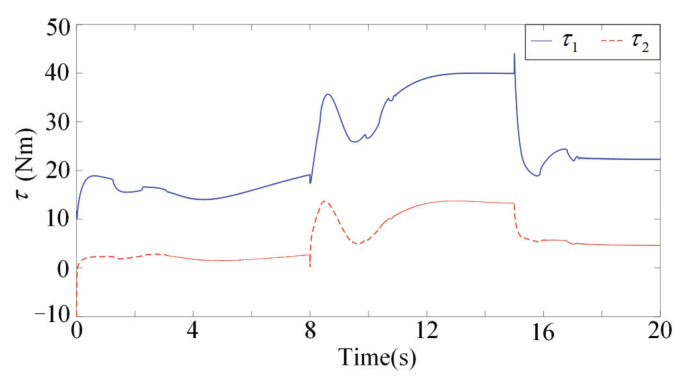
The control inputs of the finite-time multimal controller.

Figure 1, Figure 2, Figure 3 and Figure 4 present the performances of the proposed finite-time multimodal control in t∈[0,8), t∈[8,15), and t∈[15,20]. From Figure 2, the proposed multimodal control guarantees the convergence of the impedance error $e_{im}=M_{d}\left({\ddot{q}}_{d}-\ddot{q}\right)+B_{d}\left({\dot{q}}_{d}-\dot{q}\right)+K_{d}\left(q_{d}-q\right)-f_{h}$ which illustrates the achievement of the desire impedance dynamics in (6). From Figure 3 and Figure 4, the proposed multimodal control guarantees the convergence of the trajectory tracking error $e_{p}$ in t∈[8,15) and the convergence of the $\dot{q}$ to zero in t∈[15,20], which illustrates the control effectiveness in PIM and SSM. Figure 5 illustrates the continuity of the control input guaranteed by the control term ϕ in (11).

**Figure 6:**
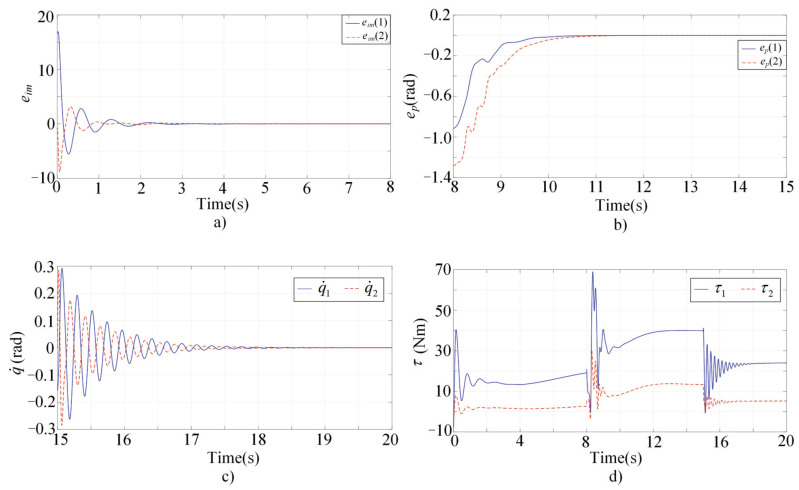
Simulationresults of the adaptive control. (**a**) The impedance errors; (**b**) The tracking errors; (**c**) The convergence of the $\dot{q}$ to zero; (**d**) The control inputs.

In order to verify the feasibility and effectiveness of the proposed method, comparisons between the proposed strategy and adaptive control have been conducted. The convergence rate and tracking error are regarded as the performance indices and compared between the two methods to provide a more comprehensive understanding of the results. The steady switch controller is neglected in the adaptive control. Performances of the adaptive control are illustrated in the Figure 6. Also, active human motion intentions are supposed to be existing in t∈[0,8), while there is no active human motion intention in t∈[8,15). At *t* = 15, the interaction is required to be stopped. The desired trajectories and desired impedances are the same as in the simulation of the proposed method.

Figure 6a shows the impedance errors in t∈[0,8). Figure 6b shows the tracking errors in t∈[8,15). The convergence of the $\dot{q}$ to zero in t∈[15,20] can be seen in Figure 6c. Figure 6d illustrates the continuity of the control input.It is found that the convergence rate of the proposed algorithm is superior compared with the adaptive control. In addition, huge improvements on tracking accuracies have been realized. Compared with Figure 6, the control oscillation of the proposed method is weakened by incorporating the steady switch control. On the whole, results show that the proposed strategy offers superior convergence rate and better tracking performance compared with the adaptive control.

## 5. Discussion

Modern robots are shown to be ubiquitous in the near future. It seems that robots will be used in all walks of life, especially in human robot co-existent scenario. People will increasingly have interactions with intelligent robots [1]. Humans may need to realize their target actions with the help of robots. With these interactions becoming deeper and deeper, it gives rise to the emerging field of research named human robot interaction [24]. A central issue in human–robot interaction is to ensure users to be safe, when the robot’s actual capabilities and reliability are often overtrusted [2]. One way to ensure users’ safety is to counteract overtrust by understanding its psychological foundations. Various studies have been conducted in this field [1,2,3]. Another way is to improve the capabilities and reliability of robots by technological innovation. In this paper, a novel finite-time interactive control algorithm is proposed for robots with multiple interaction modes. Convergence rate is of great significance in human–robot interaction, since the robot should response quickly when interacting with human in real-time. Adaptive impedance controllers have been designed by some researcher [15,25]. However, their transient performances are not satisfied. Thus, finite-time control is adopted in the control strategy. In addition, steady switch controller is designed to guarantee control input continuity and steady interaction modes transition. The capabilities and reliability of robots may be improved by the proposed method, which will enhance the safety in human robot co-existent scenario. It should be noted that counteracting overtrust and improving the capabilities and reliability of robots may be mutually reinforcing and influencing.

## 6. Conclusions

In this paper, a finite-time multi-modal robot controller is proposed for physical human–robot interaction with multiple interaction modes. The controller guarantees finite-time achievement of the control objective in each interaction mode and makes steady modes transition. We validate the finite-time control stability by Lyapunov-based theoretical analysis and illustrate the control effectiveness through simulation results. The proposed transition control term guarantees control input continuity, but it cannot ensure optimal and seamless interaction mode switches. In the coming future, optimal transition control to guarantee control continuity and seamless mode switches is one of our research interests.

## Data Availability

Not applicable.
